# Limb position affects intraoperative assessment of condylar width

**DOI:** 10.1007/s00590-023-03672-1

**Published:** 2023-08-14

**Authors:** Douglass W. Tucker, Michael J. Chen, Akhil Reddy, John J. Carney, Michael J. Gardner, Geoffrey S. Marecek

**Affiliations:** 1 Department of Orthopaedic Surgery, University of Pittsburgh Medical Center, Pittsburgh, PA USA; 2 Department of Orthopaedic Surgery, Stanford University, Redwood City, CA USA; 3 Department of Orthopaedic Surgery, Keck School of Medicine of the University of Southern California, Los Angeles, CA USA; 4 Department of Orthopaedic Surgery, Northwestern University, Chicago, IL USA; 5 Department of Orthopaedic Surgery, Cedars-Sinai Medical Center, 444 S San Vicente #603, Los Angeles, CA 90048 USA

**Keywords:** Tibial plateau, Condylar width, Limb position, Fluoroscopy, Fracture

## Abstract

**Purpose:**

We sought to define how changes in position and rotation of fluoroscopic imaging may affect the assessment of condylar widening intraoperatively.

**Methods:**

Thirty-three patients with tibial plateau fractures were prospectively identified and included in this study. Fluoroscopic images of the uninjured tibial plateau were obtained in (1) full extension and (2) slight flexion on foam ramp. Beginning with a plateau view, additional views of the tibial plateau were then obtained by rotating the fluoroscope around the knee in 5 degree increments up to 15 degrees in both internal and external rotation. Measurements of distal femoral condylar width (DFW), distal femoral articular width (FAW), proximal tibial articular width (TAW) and lateral plateau width (LPW) were performed.

**Results:**

LPW was decreased in flexion compared to extension at all degrees of rotation (*p* = 0.04–0.00001). There was a trend toward increasing LPW with increasing degrees of internal rotation which reached significance at 15˚ of internal rotation when the knee was flexed. On ANOVA, there was a significant difference of LPW with increasing degree of internal rotation when the knee was in flexion (*p* = 0.008), but not in extension. There were no differences in DFW, FAW, TAW and DFW/TAW at any point though LPW was decreased in flexion at all degrees of rotation. The FAW/TAW ratio was increased in flexion at all degrees of rotation.

**Discussion:**

The knee in flexion will underestimate the measurement of condylar width compared to the knee in full extension, by ~ 2 mm. Rotation of the knee, in comparison, did not have a significant effect on condylar width assessment.

**Level of evidence:**

Diagnostic II.

**Supplementary Information:**

The online version contains supplementary material available at 10.1007/s00590-023-03672-1.

## Introduction

The goals of treatment for tibial plateau fractures include reestablishing a neutral mechanical axis and stable knee with efficient load transfer between the femur and tibia in order to minimize the risk of post-traumatic arthrosis [1–3]. Condylar widening can affect stability and load transfer in both uni- and bi-condylar tibial plateau fractures [4, 5]. Reduction of condylar width is a crucial component during surgery as uncorrected widening is associated with worse patient outcomes [3, 6–8].

**Fig. 1:**
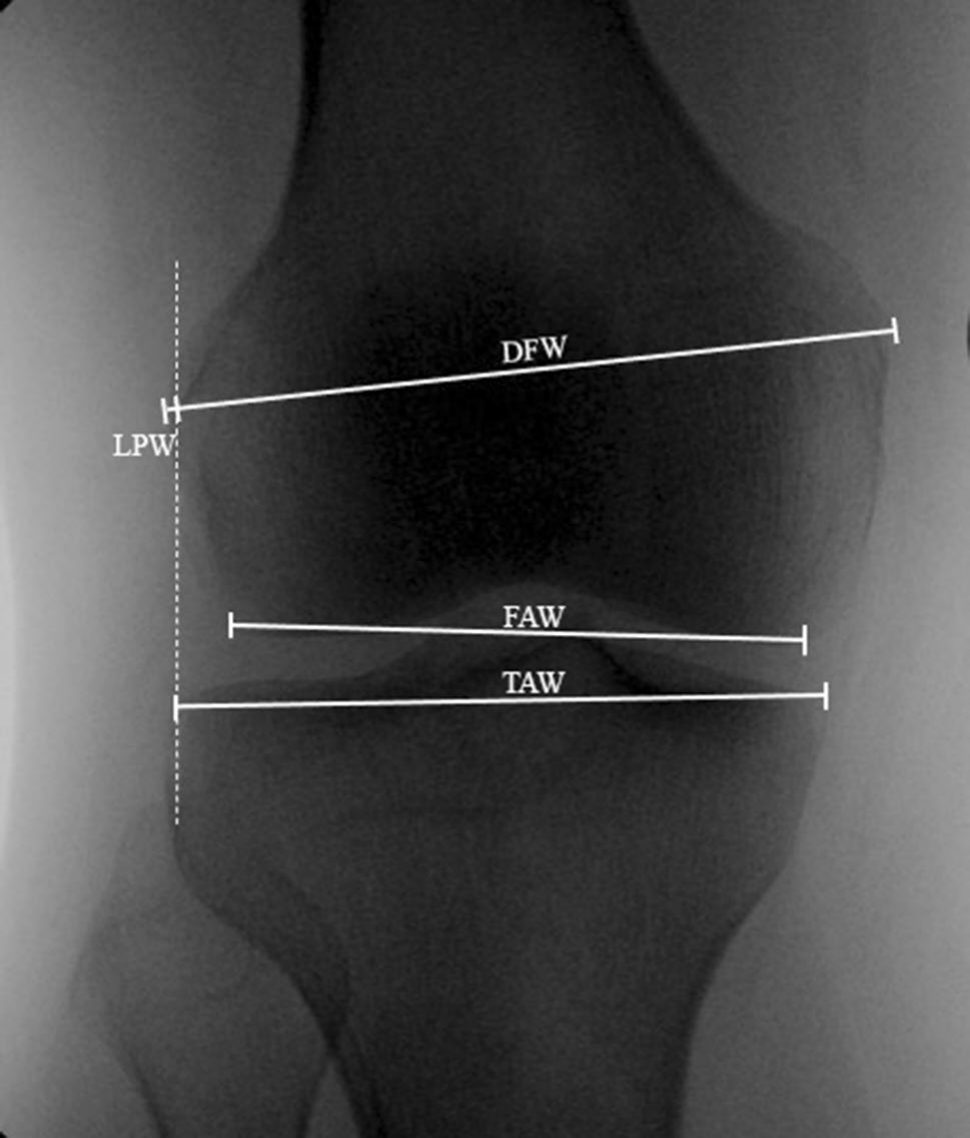
AP Radiograph with Sample of Measurements. Representative image for measurements utilized for analysis as per Johannsen et al. [2]. Distal femoral condylar width (DFW) was calculated by beginning at the medial most point of the medial femoral epicondyle and drawing line to the lateral most aspect of the lateral femoral epicondyle. The femoral articular width (FAW) was measured as a parallel from the medial femoral articular surface to the inflection point on the lateral aspect of the lateral femoral condyle. Tibial articular width (TAW) was measured parallel to the medial aspect of the medial tibial plateau articular surface to the lateral aspect of the lateral tibial articular surface. Lateral plateau width (LPW) was measured by drawing two lines perpendicular to the medial tibial articular surface, one along the most lateral aspect of the distal femoral condyle and the other along the most lateral aspect of the proximal tibia. The distance between these parallel lines was recorded. Positive values indicated that the proximal lateral tibia was more lateral than the femur, and negative values indicated the lateral femoral condyle was more lateral than the tibial plateau

Direct visualization and intraoperative fluoroscopy are both used to assess the quality of reduction during surgery. As such, reliable fluoroscopic metrics for assessing condylar width are necessary. A recent study by Johannsen et al. established normative values for tibial plateau width from a series of injured and uninjured radiographs [2]. The condylar width metrics they used, including lateral plateau widening (LPW) and the ratio of femoral articular width (FAW) to tibial articular width (TAW) are readily assessed via intraoperative fluoroscopy (Fig. 1). The contralateral, uninjured limb can be used as a viable template for assessing patient specific condylar width metrics [9–11]. Pathologic widening of the injured tibial plateau, and therefore assessment of adequate reduction, is determined by comparison to the uninjured, native anatomy.

During intraoperative radiographic evaluation, the knee may be positioned in either full extension or slight flexion depending on the use of positioning devices such as bumps or a foam ramp. However, the effect of varied limb positioning on LPW, TAW, and FAW evaluation is unknown. Variations in limb positioning has been shown to alter the reduction assessment of distal femur fractures [10]. Moreover, the leg may rotate during the surgery, creating variations in rotation. This has been shown to affect the apparent and actual starting points during tibial nailing [10, 12]. In order to achieve maximum utility of intraoperative fluoroscopy, it is essential to understand how variations in limb position affect assessment of condylar width. To our knowledge, no prior studies have examined how apparent condylar width varies with changes in limb position.

The aim of this study was to describe how changes in intraoperative limb alignment, in terms of flexion and rotation of the knee, alter fluoroscopic evaluation of condylar width. We hypothesized that increased rotation and knee flexion during fluoroscopic imaging would not affect condylar width as measured by the LPW, FAW, and TAW metrics.

## Materials and methods

Following IRB approval, we prospectively identified patients > 18 years old undergoing operative treatment of tibial plateau fractures at two level 1 trauma centers and a tertiary referral center. We excluded patients with radiographic evidence of any previous knee injury, dislocation, bony abnormalities, prior arthroplasty or prior implants. Thirty-three patients were included in this study. Patient demographics are described in Table 1. The average age was 44.3 ± 16.7 years, and the majority of patients were male (64.3%). The right knee was more commonly injured (60.7%). The most common fracture types were Schatzker II and VI (both 39.3%) [4, 5].

**Table 1:** Demographics

Demographic	Mean	Standard deviation	Median
Age	44.25	16.72	42.50
	Number	Percent	
Male	18	64.29%	
Right	11	39.29%	
Schazker classification			
II	11	39.29%	
III	1	3.57%	
IV	0	0.00%	
V	5	17.86%	
VI	11	39.29%	

### Imaging

Supine fluoroscopic images of the uninjured tibial plateau were obtained with the knee in full extension with a 1-inch calibration ball placed lateral to the knee. All images were obtained by a senior orthopaedic surgical resident, orthopaedic trauma fellow, or attending traumatologist. Beginning with a plateau view (AP) image of the proximal tibia and the flouroscopy machine perpendicular to the tibia, additional views of the tibial plateau were obtained by rotating the fluoroscopy machine around the knee in 5˚ increments up to 15˚ in both internal and external rotation [12]. This was repeated with the knee in slight flexion by elevating the leg on foam ramp (55˚) (BoneFoam, Inc, Corcoran, MN).

### Measurements

Measurements of distal femoral condylar width (DFW), FAW, TAW and LPW were performed as previously described using picture archiving and communication systems software (Synapse, Fujifilm, Stamford, CT and SECTRA, Selton, CT) (Fig. 1) [2]. A single reviewer at each institution performed the measurements with the exception of cases reviewed for inter-rater reliability. Positive LPW values indicated that the proximal lateral tibia was more lateral than the femur, and negative values indicated that the lateral femoral condyle was more lateral than the tibial plateau.

### Statistics

An a priori power analysis based on the data from Johannsen et al. determined that 26 patients would be necessary to determine a difference in LPW with alpha = 0.05 and power of 0.8. Student t-tests were used to analyze the differences between the degree of rotation (AP, 5˚, 10˚, 15˚ of internal or external rotation) and extremity orientation (flexed vs extended). Statistical analysis performed in Microsoft Excel (Microsoft, Redmond, WA) and Prism (GraphPad, San Diego CA). Additionally, ANOVA was used to evaluate differences between means at each point of rotation for each measurement within a single position of the knee. Significance was set at p < 0.05. Inter-rater reliability for each method was assessed with an intraclass correlation coefficient (ICC) with a 2- way mixed effects model and was characterized as excellent (> 0.9), good (0.75–0.9), fair (0.4–0.75), or poor (< 0.4) [13].

## Results

There were no significant differences in DFW, TAW, DFW/TAW, or FAW/TAW between the AP view and the 5˚, 10˚ and 15˚ internal/external rotation images. There was an overall trend toward increasing LPW with increasing degrees of internal rotation which reached significance at 15˚ of internal rotation when the knee was flexed (AP -0.42 ± 2.73 mm vs internal rotation 15˚ 1.03 ± 2.41 mm, *p* = 0.03) (Supplemental Table 1). On ANOVA, there was a significant difference of LPW with increasing degree of internal rotation when the knee was in flexion (*p* = 0.008), but not in extension.

**Fig. 2:**
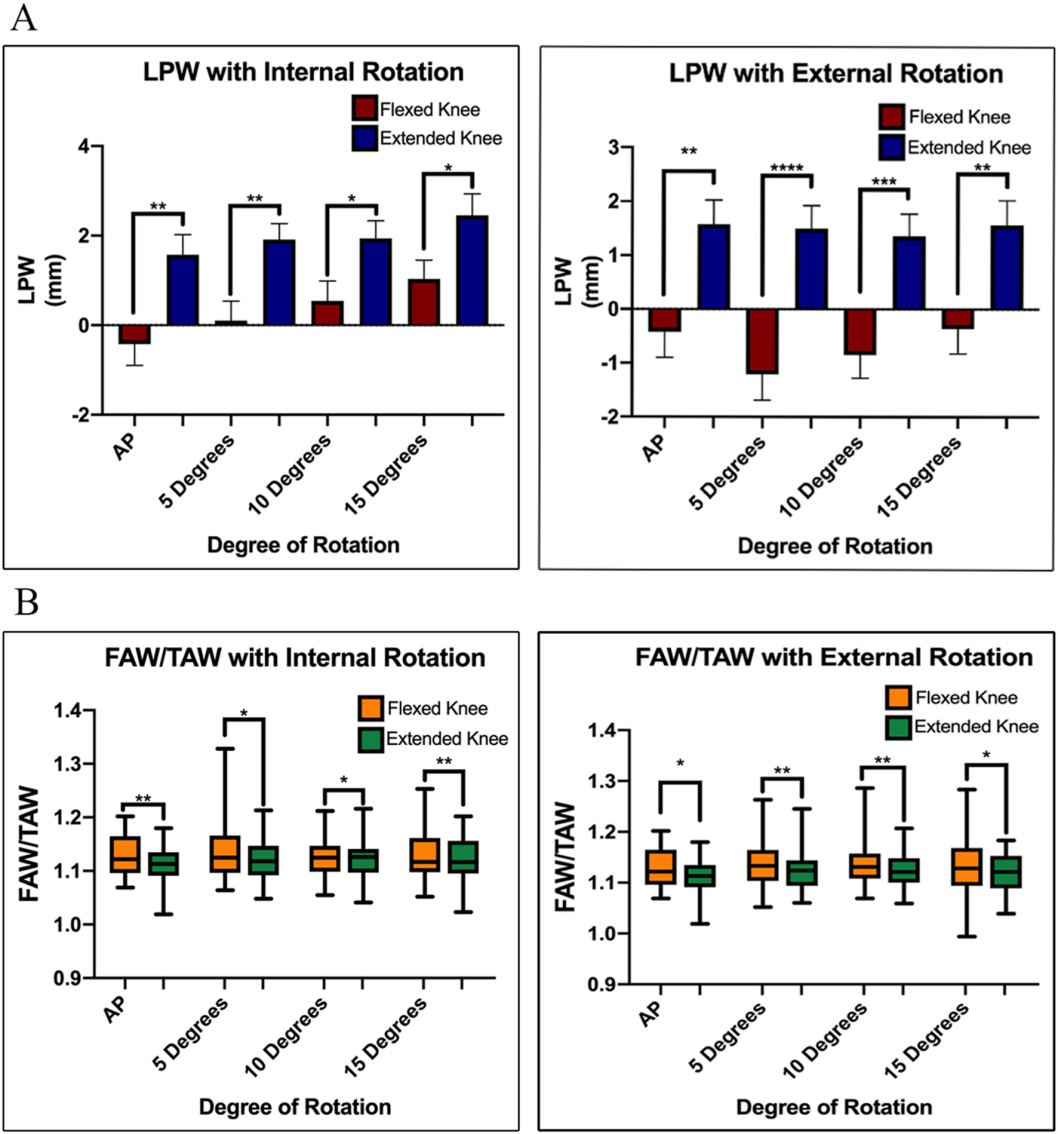
Flexion and Extension Differences in Condylar Width **A** Lateral plateau width decreases with extension at all degrees of internal and external rotation. **B** The FAW/TAW ratio decreases at all degrees of internal and external rotation. *p < 0.05, **p < 0.01, ***p < 0.001, ****p < 0.0001

There were no differences in DFW, FAW, TAW and DFW/TAW in flexion/extension at the same degree of rotation at any point. LPW was decreased in flexion compared to extension at all degrees of rotation (AP- internal rotation 15˚, AP- external rotation 15˚) (Flexion −1.21 mm–1.03 mm vs Extension 1.35 mm−2.45 mm, *p* = 0.04–0.00001) (Fig. 2). The difference between means of the knee in flexion compared to extension ranged from 1.42–2.71 mm. The FAW/TAW ratio was increased in flexion compared to extension at all degrees of rotation (flexion 0.92–0.90 vs extension 0.92–0.93, *p* = 0.03–0.009) (Table 2). The full comparison between flexion and extension at all points of rotation are described in Supplemental Table 2. Additionally, all patients had a change in LPW of 2.1 mm for at least one measurement point when comparing flexion to extension. Table 3 describes a full analysis of the number of patients with > 2.1 mm and > 5 mm of change in LPW when comparing knee position. The most common measurement to have a change greater than both 2 mm and 5 mm was internal rotation at 15 degrees (63.6% and 12.1% respectively). The lowest percentage at any alignment was internal rotation 15˚ for > 2 mm (39.4%), and external rotation 5˚ for > 5 mm (3.0%). All measurements had at least one patient with a change of > 5.21 mm (range 5.21 mm–11.82 mm). Inter-reviewer reliability was assessed for a subset of the cohort (9.1%, 3/33). Reliability was excellent for DFW, FAW and TAW (0.948–0.973) and fair for LPW (0.729) (Table 4).

**Table 2:** Flexion and extension differences

	Flexed				Extended				
AP	Average	Standard Deviation	Lower 95%	Upper 95%	Average	Standard deviation	Lower 95%	Upper 95%	P Value
LPW	− 0.42	2.73	− 1.35	0.51	1.57	2.52	0.69	2.46	0.004
FAW/TAW	0.95	0.06	0.93	0.97	0.91	0.05	0.90	0.93	0.02
External rotation 5˚									
LPW	− 1.21	2.75	− 2.15	− 0.27	1.50	2.39	0.66	2.34	< 0.0001
FAW/TAW	0.95	0.04	0.94	0.97	0.91	0.06	0.89	0.93	0.01
External rotation 10˚									
LPW	− 0.85	2.49	− 1.70	0.00	1.35	2.31	0.54	2.17	0.0005
FAW/TAW	0.95	0.06	0.93	0.97	0.91	0.05	0.89	0.93	0.009
External rotation 15˚									
LPW	− 0.36	2.62	− 1.27	0.55	1.56	2.52	0.66	2.46	0.005
FAW/TAW	0.95	0.06	0.92	0.97	0.91	0.05	0.89	0.93	0.02
Internal rotation 5˚									
LPW	− 0.19	2.51	− 1.05	0.66	1.91	1.97	1.22	2.61	0.002
FAW/TAW	0.95	0.08	0.93	0.98	0.91	0.05	0.89	0.93	0.01
Internal rotation 10˚									
LPW	0.54	2.56	− 0.33	1.41	1.94	2.16	1.16	2.71	0.02
FAW/TAW	0.94	0.06	0.92	0.96	0.91	0.05	0.89	0.93	0.03
Internal rotation 15˚									
LPW	1.03	2.41	0.21	1.86	2.45	2.61	1.52	3.39	0.03
FAW/TAW	0.94	0.08	0.92	0.97	0.90	0.05	0.88	0.92	0.01

**Table 3:** LPW changes with knee flexion versus extension

	0	ER 5	ER 10	ER 15	IR 5	IR 10	IR 15
> 2.1 mm	14 (42.2%)	17 (51.5%)	13 (39.4%)	21 (63.6%)	19 (57.6%)	16 (48.5)	13 (39.4%)
> 5 mm	2 (6.1%)	3 (9.1%)	2 (6.1%)	4 (12.1%)	2 (6.1%)	1 (3.0%)	2 (6.1%)
Maximum difference	11.82	13.68	9.34	8.86	6.49	5.21	8.08

**Table 4:** Inter-reviewer reliability

Measure	ICC	95% Confidence interval	
DFW	0.963	0.888	0.984
FAW	0.948	0.903	0.972
TAW	0.973	0.911	0.989
LPW	0.729	0.494	0.855

## Discussion

Accurate intraoperative condylar width assessment during tibial plateau fracture surgery is critical for anatomic reduction. Our study suggests that radiographic analysis of the knee in flexion underestimates the degree of condylar widening compared to a knee in full extension. Lateral plateau width was largely unaffected by rotational limb position, except in flexion and internal rotation (15 degrees). This position may be created when a ramp, bump or triangle is used, especially when an intraoperative universal distractor rests on the bump or ramp, as it tends to internally rotate the leg. The surgeon may wish to rotate the fluoroscopy unit to correct this or make assessments with the knee in extension.

Johannsen et al. demonstrated that the FAW/TAW is a useful condylar width metric in conjunction with LPW as it eliminates length calibration differences and the effects of joint congruity abnormalities [2]. Our study showed that FAW/TAW is increased during radiographic evaluation of the knee in flexion compared to extension. There were no significant changes in FAW/TAW with respect to limb rotation. We attribute the differences to the change in projection of the distal femoral anatomy on fluoroscopy. Although FAW/TAW may be more difficult to compute and interpret during intraoperative fluoroscopy compared to LPW, it is useful for plateau width assessment. The surgeon similarly may wish to place the knee in extension when making this assessment.

A recent study by Schlatterer et al. found that both femoral and tibial articular width was increased by 8 and 9.3 mm respectively in patients aged 61–80 [11]. While our study lacked the power to detect differences between age groups, this tendency towards a wider articular surface in the elderly cohort should be considered when performing reduction. However, it is important to note that our key findings revolve around ratios and measurement of the LPW. Due to the nature of our measurements (TAW, FAW, LPW), simply having a wider articular surface will not cause any changes in LPW or the FAW/TAW ratio which were increased when the knee was in flexion. With the symmetric increase of these two surfaces, there would be little change to the respective findings in patients aged 61–80.

Based on this study, the intraoperative metrics used to assess condylar width, including LPW and TAW/FAW, are dependent on knee flexion. Therefore, the position of the knee should be considered during the intraoperative fluoroscopic assessment of condylar width. The degree to which condylar width needs to be restored to achieve clinical relevance is controversial, with some authors suggesting that 5–10 mm of condylar widening is acceptable [3, 7, 14–16]. This includes a study by Honkonen et al., which found that 5 mm of condylar widening is the threshold to affect patient outcomes [3]. Barei et al. found similarly found that reducing condylar width to within 5 mm was a predictor for improved patient reported outcome scores, though they did not examine smaller differences [17]. However, more recent literature suggests that < 2 mm of widening after reduction is required to achieve an anatomic relationship [18–20]. In addition, Johannsen et al. found that condylar widening greater than 2.1 mm in lateral tibial plateau fractures is pathologic based on normative data [2].

Our study showed that differences in LPW between flexion and extension were > 1.42 mm, with certain rotations being > 2.1 mm (5˚ external rotation, 10˚ external rotation, 5˚ internal rotation). Assessing the plateau in different knee positions alters the radiographic interpretation of condylar width reduction and should be considered by the surgeon. The 2 mm difference seen in LPW with the knee in flexion vs extension can alter the assessment of condylar width reduction. To improve reduction accuracy, we recommend obtaining fluoroscopic images of the contralateral knee for comparison in the exact orientation that the operative knee will be placed during fluoroscopic assessment of the reduction. For example, if the operation is going to be performed with the leg elevated on a foam ramp with the knee in a flexed position, comparison fluoroscopic views of the contralateral side should be performed before the procedure starts in the same fashion. The contralateral tibial plateau serves as an invaluable template to guide the fluoroscopic reduction assessment, provided that the anatomy is in its native state.

The strengths of this study include its multicenter and prospective nature, and methodology for obtaining fluoroscopic plateau measurements. Our data on LPW and widening metrics were obtained from the contralateral knee in its native uninjured, and anatomic state. This increased our ability to standardize the measurements. This study is limited due to all measurements being performed by a single reviewer at their respective institution. Thus, we are unable to perform inter/intra reviewer reliability for all patients in the study, though we do have a small cohort for which this was examined. Additionally other studies using similar measurement techniques have reported excellent correlation [2, 9]. This study found that performing intraoperative fluoroscopic assessment of the tibial plateau with the knee in flexion will underestimate the measurement of condylar width, compared to the knee in full extension, by ~ 2 mm. This is important as 2 mm is regarded by some studies as the cutoff for adequate reduction to improve clinical outcomes. Rotation of the knee, in comparison, did not have a significant effect on condylar width assessment. We recommend obtaining contralateral fluoroscopic images with the uninjured knee placed in the same position to serve as a template for reduction assessment. Surgeons should be aware of these variations and interpret imaging accordingly.

## Conclusions

Measurement of condylar width is significantly affected by the flexion or extension of the knee. However, the rotational profile for radiographs does not significantly affect the condylar width. When comparing an injured radiograph to the contralateral side to ensure symmetric reduction, care must be taken to ensure that orientation is consistent between the injured and uninjured side especially when evaluating for changes in lateral plateau width.

### Supplementary Information

Below is the link to the electronic supplementary material.

Supplemental Table 1: Each measurement at all recorded degrees of rotation compared to AP radiographs (DOCX 27 KB)

Supplemental Table 2: All measurements comparing flexion to extension in a given rotation plane. (DOCX 27 KB)
