# Supplementary material for: Limb position affects intraoperative assessment of condylar width
Source: Eur J Orthop Surg Traumatol. 2023 Aug 14;34(1):451–7. doi: 10.1007/s00590-023-03672-1 (PMC10771358; doi:10.1007/s00590-023-03672-1)
Supplement: Supplementary file 1 — Supplemental Table 1: Each measurement at all recorded degrees of rotation compared to AP radiographs (DOCX 27 KB) [file 590_2023_3672_MOESM1_ESM.docx]

Supplementary Table 1

| DFW | | | | | |  | FAW | | | | | |
| --- | --- | --- | --- | --- | --- | --- | --- | --- | --- | --- | --- | --- |
|  | **Flexed** | | | | |  |  | **Flexed** | | | | |
|  | **Average** | **Standard Deviation** | **Lower 95%** | **Upper 95%** | **P Value** |  |  | **Average** | **Standard Deviation** | **Lower 95%** | **Upper 95%** | **P Value** |
| AP | 83.05 | 8.94 | 80.00 | 86.10 | NA |  | AP | 69.70 | 8.89 | 66.67 | 72.73 | NA |
| External Rotation 5˚ | 82.99 | 8.56 | 80.07 | 85.91 | 0.97 |  | External Rotation 5˚ | 69.60 | 8.87 | 66.57 | 72.62 | 0.96 |
| External Rotation 10˚ | 82.23 | 9.05 | 79.14 | 85.31 | 0.71 |  | External Rotation 10˚ | 68.46 | 9.60 | 65.18 | 71.74 | 0.59 |
| External Rotation 15˚ | 82.14 | 9.41 | 78.88 | 85.40 | 0.69 |  | External Rotation 15˚ | 68.56 | 9.76 | 65.18 | 71.94 | 0.62 |
| Internal Rotation 5˚ | 84.12 | 8.72 | 81.14 | 87.09 | 0.63 |  | Internal Rotation 5˚ | 72.90 | 8.89 | 69.86 | 75.93 | 0.88 |
| Internal Rotation 10˚ | 83.62 | 8.27 | 80.80 | 86.44 | 0.79 |  | Internal Rotation 10˚ | 69.71 | 8.39 | 66.84 | 72.57 | 0.99 |
| Internal Rotation 15˚ | 83.63 | 9.08 | 80.54 | 86.73 | 0.79 |  | Internal Rotation 15˚ | 69.63 | 9.30 | 66.46 | 72.81 | 0.98 |
|  | **Extended** | | | | |  |  | **Extended** | | | | |
|  | **Average** | **Standard Deviation** | **Lower 95%** | **Upper 95%** | **P Value** |  |  | **Average** | **Standard Deviation** | **Lower 95%** | **Upper 95%** | **P Value** |
| AP | 82.67 | 8.46 | 79.69 | 85.65 | NA |  | AP | 68.25 | 8.46 | 65.27 | 71.22 | NA |
| External Rotation 5˚ | 82.66 | 8.93 | 79.51 | 85.80 | 0.99 |  | External Rotation 5˚ | 67.49 | 8.84 | 64.38 | 70.60 | 0.73 |
| External Rotation 10˚ | 82.44 | 8.59 | 79.42 | 85.47 | 0.92 |  | External Rotation 10˚ | 67.04 | 8.73 | 63.97 | 70.12 | 0.58 |
| External Rotation 15˚ | 81.41 | 9.42 | 78.04 | 84.78 | 0.58 |  | External Rotation 15˚ | 66.36 | 9.03 | 63.13 | 69.59 | 0.4 |
| Internal Rotation 5˚ | 83.18 | 8.13 | 80.32 | 86.04 | 0.81 |  | Internal Rotation 5˚ | 67.65 | 7.95 | 64.85 | 70.45 | 0.78 |
| Internal Rotation 10˚ | 83.41 | 8.48 | 80.42 | 86.39 | 0.73 |  | Internal Rotation 10˚ | 67.81 | 8.45 | 64.83 | 70.78 | 0.84 |
| Internal Rotation 15˚ | 82.66 | 8.09 | 79.76 | 85.55 | 0.99 |  | Internal Rotation 15˚ | 66.60 | 7.78 | 63.82 | 69.39 | 0.43 |
|  |  |  |  |  |  |  |  |  |  |  |  |  |
| TAW | | | | | |  | LPW | | | | | |
|  | **Flexed** | | | | |  |  | **Flexed** | | | | |
|  | **Average** | **Standard Deviation** | **Lower 95%** | **Upper 95%** | **P Value** |  |  | **Average** | **Standard Deviation** | **Lower 95%** | **Upper 95%** | **P Value** |
| AP | 73.84 | 8.75 | 70.85 | 76.82 | NA |  | AP | -0.42 | 2.73 | -1.35 | 0.51 | NA |
| External Rotation 5˚ | 73.34 | 8.85 | 70.32 | 76.36 | 0.81 |  | External Rotation 5˚ | -1.21 | 2.75 | -2.15 | -0.27 | 0.25 |
| External Rotation 10˚ | 72.50 | 8.82 | 69.49 | 75.51 | 0.54 |  | External Rotation 10˚ | -0.85 | 2.49 | -1.70 | 0.00 | 0.51 |
| External Rotation 15˚ | 72.83 | 9.18 | 69.64 | 76.01 | 0.65 |  | External Rotation 15˚ | -0.36 | 2.62 | -1.27 | 0.55 | 0.93 |
| Internal Rotation 5˚ | 75.91 | 8.25 | 73.10 | 78.72 | 0.92 |  | Internal Rotation 5˚ | -0.19 | 2.51 | -1.05 | 0.66 | 0.42 |
| Internal Rotation 10˚ | 74.44 | 7.77 | 71.79 | 77.09 | 0.77 |  | Internal Rotation 10˚ | 0.54 | 2.56 | -0.33 | 1.41 | 0.14 |
| Internal Rotation 15˚ | 74.21 | 8.44 | 71.33 | 77.09 | 0.86 |  | Internal Rotation 15˚ | 1.03 | 2.41 | 0.21 | 1.86 | **0.03** |
|  | **Extended** | | | | |  |  | **Extended** | | | | |
|  | **Average** | **Standard Deviation** | **Lower 95%** | **Upper 95%** | **P Value** |  |  | **Average** | **Standard Deviation** | **Lower 95%** | **Upper 95%** | **P Value** |
| AP | 74.39 | 8.12 | 71.53 | 77.25 | NA |  | AP | 1.57 | 2.52 | 0.69 | 2.46 | NA |
| External Rotation 5˚ | 73.82 | 8.70 | 70.76 | 76.88 | 0.79 |  | External Rotation 5˚ | 1.50 | 2.39 | 0.66 | 2.34 | 0.9 |
| External Rotation 10˚ | 73.59 | 8.66 | 70.54 | 76.64 | 0.71 |  | External Rotation 10˚ | 1.35 | 2.31 | 0.54 | 2.17 | 0.72 |
| External Rotation 15˚ | 72.78 | 9.32 | 69.44 | 76.11 | 0.47 |  | External Rotation 15˚ | 1.56 | 2.52 | 0.66 | 2.46 | 0.99 |
| Internal Rotation 5˚ | 74.46 | 8.10 | 71.61 | 77.31 | 0.97 |  | Internal Rotation 5˚ | 1.91 | 1.97 | 1.22 | 2.61 | 0.55 |
| Internal Rotation 10˚ | 74.47 | 8.73 | 71.40 | 77.55 | 0.97 |  | Internal Rotation 10˚ | 1.94 | 2.16 | 1.16 | 2.71 | 0.18 |
| Internal Rotation 15˚ | 74.08 | 8.32 | 71.11 | 77.06 | 0.88 |  | Internal Rotation 15˚ | 2.45 | 2.61 | 1.52 | 3.39 | 0.55 |
|  |  |  |  |  |  |  |  |  |  |  |  |  |
| DFW/TAW | | | | | |  | FAW/TAW | | | | | |
|  | **Flexed** | | | | |  |  | **Flexed** | | | | |
|  | **Average** | **Standard Deviation** | **Lower 95%** | **Upper 95%** | **P Value** |  |  | **Average** | **Standard Deviation** | **Lower 95%** | **Upper 95%** | **P Value** |
| AP | 1.13 | 0.04 | 1.11 | 1.14 | NA |  | AP | 0.95 | 0.06 | 0.93 | 0.97 | NA |
| External Rotation 5˚ | 1.13 | 0.04 | 1.12 | 1.15 | 0.43 |  | External Rotation 5˚ | 0.95 | 0.04 | 0.94 | 0.97 | 0.86 |
| External Rotation 10˚ | 1.14 | 0.04 | 1.12 | 1.15 | 0.33 |  | External Rotation 10˚ | 0.95 | 0.06 | 0.93 | 0.97 | 0.87 |
| External Rotation 15˚ | 1.13 | 0.06 | 1.11 | 1.15 | 0.73 |  | External Rotation 15˚ | 0.95 | 0.06 | 0.92 | 0.97 | 0.75 |
| Internal Rotation 5˚ | 1.14 | 0.05 | 1.12 | 1.16 | 0.32 |  | Internal Rotation 5˚ | 0.95 | 0.08 | 0.93 | 0.98 | 0.78 |
| Internal Rotation 10˚ | 1.12 | 0.04 | 1.11 | 1.14 | 0.82 |  | Internal Rotation 10˚ | 0.94 | 0.06 | 0.92 | 0.96 | 0.52 |
| Internal Rotation 15˚ | 1.13 | 0.04 | 1.11 | 1.14 | 0.87 |  | Internal Rotation 15˚ | 0.94 | 0.08 | 0.92 | 0.97 | 0.78 |
|  | **Extended** | | | | |  |  | **Extended** | | | | |
|  | **Average** | **Standard Deviation** | **Lower 95%** | **Upper 95%** | **P Value** |  |  | **Average** | **Standard Deviation** | **Lower 95%** | **Upper 95%** | **P Value** |
| AP | 1.11 | 0.04 | 1.10 | 1.13 | NA |  | AP | 0.91 | 0.05 | 0.90 | 0.93 | NA |
| External Rotation 5˚ | 1.12 | 0.04 | 1.11 | 1.14 | 0.35 |  | External Rotation 5˚ | 0.91 | 0.06 | 0.89 | 0.93 | 0.85 |
| External Rotation 10˚ | 1.12 | 0.04 | 1.11 | 1.14 | 0.3 |  | External Rotation 10˚ | 0.91 | 0.05 | 0.89 | 0.93 | 0.61 |
| External Rotation 15˚ | 1.12 | 0.04 | 1.11 | 1.13 | 0.39 |  | External Rotation 15˚ | 0.91 | 0.05 | 0.89 | 0.93 | 0.7 |
| Internal Rotation 5˚ | 1.12 | 0.04 | 1.11 | 1.13 | 0.5 |  | Internal Rotation 5˚ | 0.91 | 0.05 | 0.89 | 0.93 | 0.54 |
| Internal Rotation 10˚ | 1.12 | 0.04 | 1.11 | 1.14 | 0.31 |  | Internal Rotation 10˚ | 0.91 | 0.05 | 0.89 | 0.93 | 0.61 |
| Internal Rotation 15˚ | 1.12 | 0.04 | 1.10 | 1.13 | 0.59 |  | Internal Rotation 15˚ | 0.90 | 0.05 | 0.88 | 0.92 | 0.2 |
