# Supplementary material for: Limb position affects intraoperative assessment of condylar width
Source: Eur J Orthop Surg Traumatol. 2023 Aug 14;34(1):451–7. doi: 10.1007/s00590-023-03672-1 (PMC10771358; doi:10.1007/s00590-023-03672-1)
Supplement: Supplementary file 2 — Supplemental Table 2: All measurements comparing flexion to extension in a given rotation plane. (DOCX 27 KB) [file 590_2023_3672_MOESM2_ESM.docx]

Supplementary Table 2

|  |  | **Flexed** | | | |  | **Extended** | | | |  |
| --- | --- | --- | --- | --- | --- | --- | --- | --- | --- | --- | --- |
| **AP** | **Measurement** | **Average** | **Standard Deviation** | **Lower 95%** | **Upper 95%** |  | **Average** | **Standard Deviation** | **Lower 95%** | **Upper 95%** | **P Value** |
|  | **DFW** | 83.05 | 8.94 | 80.00 | 86.10 |  | 82.67 | 8.46 | 79.69 | 85.65 | 0.86 |
|  | **FAW** | 69.70 | 8.89 | 66.67 | 72.73 |  | 68.25 | 8.46 | 65.27 | 71.22 | 0.5 |
|  | **TAW** | 73.84 | 8.75 | 70.85 | 76.82 |  | 74.39 | 8.12 | 71.53 | 77.25 | 0.8 |
|  | **LPW** | -0.42 | 2.73 | -1.35 | 0.51 |  | 1.57 | 2.52 | 0.69 | 2.46 | 0.004 |
|  | **DFW/TAW** | 1.13 | 0.04 | 1.11 | 1.14 |  | 1.11 | 0.04 | 1.10 | 1.13 | 0.13 |
|  | **FAW/TAW** | 0.95 | 0.06 | 0.93 | 0.97 |  | 0.91 | 0.05 | 0.90 | 0.93 | 0.02 |
|  |  |  |  |  |  |  |  |  |  |  |  |
| **External Rotation 5** | **Measurement** | **Average** | **Standard Deviation** | **Lower 95%** | **Upper 95%** |  | **Average** | **Standard Deviation** | **Lower 95%** | **Upper 95%** | **P Value** |
|  | **DFW** | 82.99 | 8.56 | 80.07 | 85.91 |  | 82.66 | 8.93 | 79.51 | 85.80 | 0.88 |
|  | **FAW** | 69.60 | 8.87 | 66.57 | 72.62 |  | 67.49 | 8.84 | 64.38 | 70.60 | 0.35 |
|  | **TAW** | 73.34 | 8.85 | 70.32 | 76.36 |  | 73.82 | 8.70 | 70.76 | 76.88 | 0.83 |
|  | **LPW** | -1.21 | 2.75 | -2.15 | -0.27 |  | 1.50 | 2.39 | 0.66 | 2.34 | <0.0001 |
|  | **DFW/TAW** | 1.13 | 0.04 | 1.12 | 1.15 |  | 1.12 | 0.04 | 1.11 | 1.14 | 0.22 |
|  | **FAW/TAW** | 0.95 | 0.04 | 0.94 | 0.97 |  | 0.91 | 0.06 | 0.89 | 0.93 | 0.01 |
|  |  |  |  |  |  |  |  |  |  |  |  |
| **External Rotation 10** | **Measurement** | **Average** | **Standard Deviation** | **Lower 95%** | **Upper 95%** |  | **Average** | **Standard Deviation** | **Lower 95%** | **Upper 95%** | **P Value** |
|  | **DFW** | 82.23 | 9.05 | 79.14 | 85.31 |  | 82.44 | 8.59 | 79.42 | 85.47 | 0.92 |
|  | **FAW** | 68.46 | 9.60 | 65.18 | 71.74 |  | 67.04 | 8.73 | 63.97 | 70.12 | 0.54 |
|  | **TAW** | 72.50 | 8.82 | 69.49 | 75.51 |  | 73.59 | 8.66 | 70.54 | 76.64 | 0.62 |
|  | **LPW** | -0.85 | 2.49 | -1.70 | 0.00 |  | 1.35 | 2.31 | 0.54 | 2.17 | 0.0005 |
|  | **DFW/TAW** | 1.14 | 0.04 | 1.12 | 1.15 |  | 1.12 | 0.04 | 1.11 | 1.14 | 0.17 |
|  | **FAW/TAW** | 0.95 | 0.06 | 0.93 | 0.97 |  | 0.91 | 0.05 | 0.89 | 0.93 | 0.009 |
|  |  |  |  |  |  |  |  |  |  |  |  |
| **External Rotation 15** | **Measurement** | **Average** | **Standard Deviation** | **Lower 95%** | **Upper 95%** |  | **Average** | **Standard Deviation** | **Lower 95%** | **Upper 95%** | **P Value** |
|  | **DFW** | 82.14 | 9.41 | 78.88 | 85.40 |  | 81.41 | 9.42 | 78.04 | 84.78 | 0.76 |
|  | **FAW** | 68.56 | 9.76 | 65.18 | 71.94 |  | 66.36 | 9.03 | 63.13 | 69.59 | 0.36 |
|  | **TAW** | 72.83 | 9.18 | 69.64 | 76.01 |  | 72.78 | 9.32 | 69.44 | 76.11 | 0.98 |
|  | **LPW** | -0.36 | 2.62 | -1.27 | 0.55 |  | 1.56 | 2.52 | 0.66 | 2.46 | 0.005 |
|  | **DFW/TAW** | 1.13 | 0.06 | 1.11 | 1.15 |  | 1.12 | 0.04 | 1.11 | 1.13 | 0.42 |
|  | **FAW/TAW** | 0.95 | 0.06 | 0.92 | 0.97 |  | 0.91 | 0.05 | 0.89 | 0.93 | 0.02 |
|  |  |  |  |  |  |  |  |  |  |  |  |
| **Internal Rotation 5** | **Measurement** | **Average** | **Standard Deviation** | **Lower 95%** | **Upper 95%** |  | **Average** | **Standard Deviation** | **Lower 95%** | **Upper 95%** | **P Value** |
|  | **DFW** | 84.12 | 8.72 | 81.14 | 87.09 |  | 83.18 | 8.13 | 80.32 | 86.04 | 0.66 |
|  | **FAW** | 72.90 | 8.89 | 69.86 | 75.93 |  | 67.65 | 7.95 | 64.85 | 70.45 | 0.26 |
|  | **TAW** | 75.91 | 8.25 | 73.10 | 78.72 |  | 74.46 | 8.10 | 71.61 | 77.31 | 0.84 |
|  | **LPW** | -0.19 | 2.51 | -1.05 | 0.66 |  | 1.91 | 1.97 | 1.22 | 2.61 | 0.002 |
|  | **DFW/TAW** | 1.14 | 0.05 | 1.12 | 1.16 |  | 1.12 | 0.04 | 1.11 | 1.13 | 0.11 |
|  | **FAW/TAW** | 0.95 | 0.08 | 0.93 | 0.98 |  | 0.91 | 0.05 | 0.89 | 0.93 | 0.01 |
|  |  |  |  |  |  |  |  |  |  |  |  |
| **Internal Rotation 10** | **Measurement** | **Average** | **Standard Deviation** | **Lower 95%** | **Upper 95%** |  | **Average** | **Standard Deviation** | **Lower 95%** | **Upper 95%** | **P Value** |
|  | **DFW** | 83.62 | 8.27 | 80.80 | 86.44 |  | 83.41 | 8.48 | 80.42 | 86.39 | 0.92 |
|  | **FAW** | 69.71 | 8.39 | 66.84 | 72.57 |  | 67.81 | 8.45 | 64.83 | 70.78 | 0.37 |
|  | **TAW** | 74.44 | 7.77 | 71.79 | 77.09 |  | 74.47 | 8.73 | 71.40 | 77.55 | 0.99 |
|  | **LPW** | 0.54 | 2.56 | -0.33 | 1.41 |  | 1.94 | 2.16 | 1.16 | 2.71 | 0.02 |
|  | **DFW/TAW** | 1.12 | 0.04 | 1.11 | 1.14 |  | 1.12 | 0.04 | 1.11 | 1.14 | 0.83 |
|  | **FAW/TAW** | 0.94 | 0.06 | 0.92 | 0.96 |  | 0.91 | 0.05 | 0.89 | 0.93 | 0.03 |
|  |  |  |  |  |  |  |  |  |  |  |  |
| **Internal Rotation 15** | **Measurement** | **Average** | **Standard Deviation** | **Lower 95%** | **Upper 95%** |  | **Average** | **Standard Deviation** | **Lower 95%** | **Upper 95%** | **P Value** |
|  | **DFW** | 83.63 | 9.08 | 80.54 | 86.73 |  | 82.66 | 8.09 | 79.76 | 85.55 | 0.66 |
|  | **FAW** | 69.63 | 9.30 | 66.46 | 72.81 |  | 66.60 | 7.78 | 63.82 | 69.39 | 0.17 |
|  | **TAW** | 74.21 | 8.44 | 71.33 | 77.09 |  | 74.08 | 8.32 | 71.11 | 77.06 | 0.95 |
|  | **LPW** | 1.03 | 2.41 | 0.21 | 1.86 |  | 2.45 | 2.61 | 1.52 | 3.39 | 0.03 |
|  | **DFW/TAW** | 1.13 | 0.04 | 1.11 | 1.14 |  | 1.12 | 0.04 | 1.10 | 1.13 | 0.35 |
|  | **FAW/TAW** | 0.94 | 0.08 | 0.92 | 0.97 |  | 0.90 | 0.05 | 0.88 | 0.92 | 0.01 |
